# Determining reference ranges for immunological cells of healthy indigenous individuals from a region in Brazil

**DOI:** 10.31744/einstein_journal/2023AO0291

**Published:** 2023-10-10

**Authors:** Isa Rita Brito de Morais, Dyjaene de Oliveira Barbosa, Gabriel Barroso de Almeida, Regina Rossoni da Costa, Bruna Oliveira da Silva, Laís Albuquerque de Oliveira, Julia Pimentel Arantes, Layla Oliveira Campos Leite, Luana Rossato, Marcos Borges Ribeiro, Silvana Beutinger Marchioro, Songelí Menezes Freire, Roberto José Meyer Nascimento, Simone Simionatto, Alex José Leite Torres

**Affiliations:** 1 Universidade Federal da Bahia Salvador BA Brazil Universidade Federal da Bahia , Salvador , BA , Brazil .; 2 Universidade Federal da Grande Dourados Dourados MS Brazil Universidade Federal da Grande Dourados , Dourados , MS , Brazil .

**Keywords:** Reference values, Indigenous peoples, Lymphocytes, Monocytes, Antibodies, monoclonal, Immune system, Health services, indigenous, Brazil

## Abstract

**Objective:**

The establishment of reference values for a subset of leukocytes is common in clinical practice, and ethnic variations are strongly associated with disease development. In Brazil, indigenous people are vulnerable to infections, and few studies have described the health and disease conditions of this population. This study aimed to provide reference values for immunological cell subsets in indigenous Brazilians living in the state of Mato Grosso do Sul.

**Methods:**

Flow cytometry and 4-color combinations of monoclonal antibodies were used to characterize cells. A total of 115 healthy adults, mostly females (72%), were included in the study. The results are presented as mean and median (2.5%-97.5% percentiles) for T and B lymphocytes, CD4+ T cells, CD8+ T cells, Natural Killer cells, monocytes, and dendritic cells, providing an average immunological profile for the population in question.

**Results:**

The relative medians of CD3+, CD4+, and CD8+ T cells were significantly higher in women than in men in a healthy indigenous population.

**Conclusion:**

To our knowledge, cell reference data from indigenous Brazilians are unknown in the literature. The immune cell results presented in this pioneering study will contribute to the clinical and laboratory evaluation of the Brazilian indigenous population, especially given the important differences when compared with other Brazilian ethnic groups.

## In Brief

Morais et al. conducted a pioneering study with Brazilian indigenous populations to determine reference values for immunologic cells from healthy adult individuals. The main findings included a higher relative median for T lymphocyte subsets in females than males, and T CD3+, T CD4+, and T CD8+ relative values were statistically different when compared with Brazilian populations from other Brazilian regions.

## Highlights

The relative medians of CD3+, CD4+, and CD8+ T cells were significantly higher in women than in men in a healthy indigenous population.

Demographic and ethnic diversity of the Brazilian population can be associated with quantitative modifications in the immunologic cells of healthy individuals.

## Visual Abstract

**Figure:**
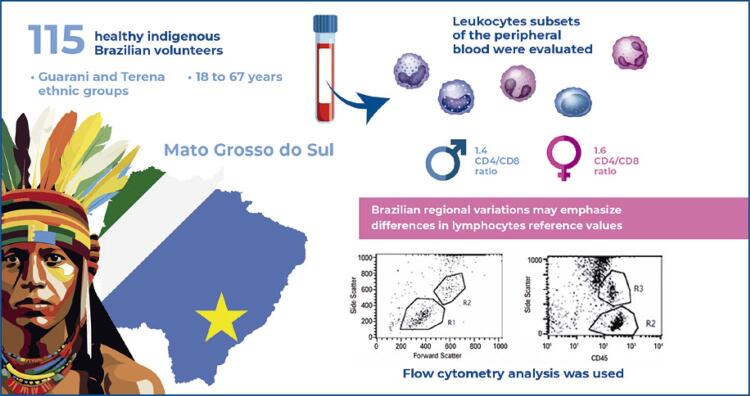

## INTRODUCTION

The reference range for leukocyte subsets is commonly used in clinical practice to diagnose various conditions, including disease progression, clinical staging, epidemiological studies, and infection prevention. ^( 1 , 2 )^ Studies on the pathogenesis of the human immunodeficiency virus (HIV) and its progression to acquired immunodeficiency syndrome (AIDS) have attracted increased interest because lymphocyte subsets are significant targets of the virus. ^( 3 )^

In the last decade, some studies have sought to determine cellular reference ranges in different parts of the world because factors that cause variations between population groups have been identified. ^( 4 )^ Geographical locations, dietary habits, ethnic variations, and environmental and climatic factors were documented as interfering variables for uniqueness from these cell values. ^( 5 , 6 )^

Laboratory reference values are needed to assess worsening preexisting conditions, the occurrence of new conditions, adverse reactions to vaccines, and toxicity related to investigational products in volunteers participating in clinical trials and in patients evaluated by physicians during clinical practice. ^( 7 )^

Ethnic variations are strongly associated with the development of diseases, such as cardiovascular diseases, atherosclerosis, Parkinson’s disease, alcohol metabolism, multiple sclerosis, viral infections, and hematologic diseases. ^( 8 - 12 )^ Hispanic/Latino individuals are more likely to have controlled risk factors for cardiovascular disease than African Americans or Caucasians. ^( 13 )^

Indigenous people are among the most vulnerable populations in Brazil and are often underrepresented in studies on the relationship between disease and health. Indigenous Brazilians constitute one of the largest indigenous populations globally, with 1.108,970 people categorized into 230 ethnic groups and over 180 languages. ^( 14 - 16 )^ Mato Grosso do Sul is a Brazilian state with the second-largest indigenous population in the country, living socially with non-indigenous people, which may increase the spread of COVID-19 in this population).

Efforts to address the health disparities and unfavorable conditions faced by indigenous populations are crucial to improve the management of prevalent diseases, guide treatments, and enhance prognosis. ^( 17 )^ Scientific publications or relevant information about these population groups are scarce, ^( 16 , 18 )^ and cellular parameters are important indicators for assessing the clinical and laboratory conditions of indigenous people.

Although they have had more comprehensive access to health services since the creation of the Indigenous Health Care Subsystem (SasiSUS - *Subsistema de Atenção à Saúde Indígena do* SUS) in the Unified Health System (SUS - *Sistema Único de Saúde* ), indigenous conditions still need to be improved to ensure good health for users.

Health indicators, such as reference values for blood cells, are important parameters for the clinical evaluation of individuals. The proposal presented in this work represents a pioneering effort in the study of immune cells in Brazilian Indians.

## OBJECTIVE

To analyze and define reference values for lymphocytes, monocyte subsets, and dendritic cells associated with epidemiological factors, such as age, sex, and lifestyle, in healthy indigenous volunteers from the city of Dourados, Mato Grosso do Sul, Brazil.

## METHODS

### Study design, setting, and population

A cross-sectional study was conducted on indigenous Brazilians from different villages in the city of Dourados, Mato Grosso do Sul, Brazil. We included 115 healthy adults defined by the absence of any clinical symptoms, previous undisclosed diseases, no pharmacological therapy, and non-reactivity to COVID-19 serology.

The study participants were healthy, eligible adults aged over 18 years who agreed to participate after providing written informed consent. Peripheral blood samples were collected in tubes containing ethylenediaminetetraacetic acid (EDTA) tubes for immunophenotyping.

A structured questionnaire containing sociodemographic information was administered to each volunteer. Individuals undergoing medical treatment, pregnant women, or those with a reactive serological diagnosis were excluded from the study.

### Laboratory procedure

Flow cytometry immunophenotyping was performed to determine the cell profile using monoclonal antibodies labeled with fluorescein isothiocyanate (FITC) fluorophore, phycoerythrin fluorophore (PE), and peridinin chlorophyll protein (PerCP) for cell surface and cytoplasmic receptor identification. All monoclonal antibodies were purchased from Becton, Dickinson & Company.

Leukocytes of the adaptative immune response were analyzed for T helper lymphocytes to CD3+(FITC) CD4+(PE) CD45+(PerCP), cytotoxic T cells with CD3+(FITC) CD8+(PE) CD45+(PerCP), double-positive T cells with CD3+ (FITC) CD4+ (PE) CD8+(APC), B cells identified by CD5+(FITC) CD19+(PE) CD45+ (PerCP) and regulatory T cells characterized by CD4+(FITC) CD25+high(PE) FoxP3(PerCP). Naïve cells were identified as CD45RA+ (FITC) CD3+(PE) CD45+(PerCP); Natural Killer cells as CD16+(FITC) CD56+(PE) CD45+(PerCP); and dendritic cells as CD11c (FITC) CD40+(PE). Monocyte subsets were determined based on CD16+ and CD14+ surface membrane expression. CD14+bright CD16- expression was determined according to classical monocytes, non-classical monocytes by CD14+dim CD16+bright profile, and intermediate monocytes by CD14+bright and CD16+dim. CD14 and CD16 monoclonal antibodies were labeled with FITC and PE, respectively.

Each cell type was processed in a tube, and monoclonal antibodies were added and incubated with 100µL of whole blood according to basic flow cytometry protocols. Samples were acquired from approximately one hundred thousand events and analyzed on a FACSCalibur (Becton Dickinson) using the CellQuest software.

Additionally, we compared the values for CD3+ T, CD4+ T, and CD8+T lymphocytes in the samples from the present study with the results established and published in our previous study in 2013 ^( 19 )^ using samples from healthy individuals from five different Brazilian states, one from each region of the country.

### Data management and statistical analysis

Completed questionnaires were coded with patient numbers and initials. Data were analyzed using SPSS version 22 software (IBM Corp, Armonk, NY, USA). The χ ^2^ test was used for categorical variables. We calculated the mean and standard deviation, 2.5 ^th^ -97.5 ^th^ percentile for the median, and odds ratios (OR) with 95% confidence intervals (CI). Comparisons between continuous variables were performed using analysis of variance or the Kruskal-Wallis test. A p<0.05 was considered statistically significant.

### Ethics

The study was approved by the Research Ethics Committee of *Universidade Federal da Grande Dourados* (CAAE: 38981720.5.1001.5160; # 4.584.624) and conducted according to Resolution 466 from December 2012 and the Helsinki Declaration of 1975, revised in 1983. Informed consent was obtained from all volunteers.

## RESULTS

### Characteristics of the study participants

A total of 115 indigenous Brazilian volunteers were included in this study. The individuals belonged to the Guarani and Terena ethnic groups, with ages ranging from 18 to 67 years. Most adults (72%) were female, with a mean age of 38 years (interquartile range, 19-51 years). Twenty-nine individuals (26%) reported smoking, and 30 (26.6%) reported drinking alcohol three or more days a week.

### Gating strategy

**Figure 1:**
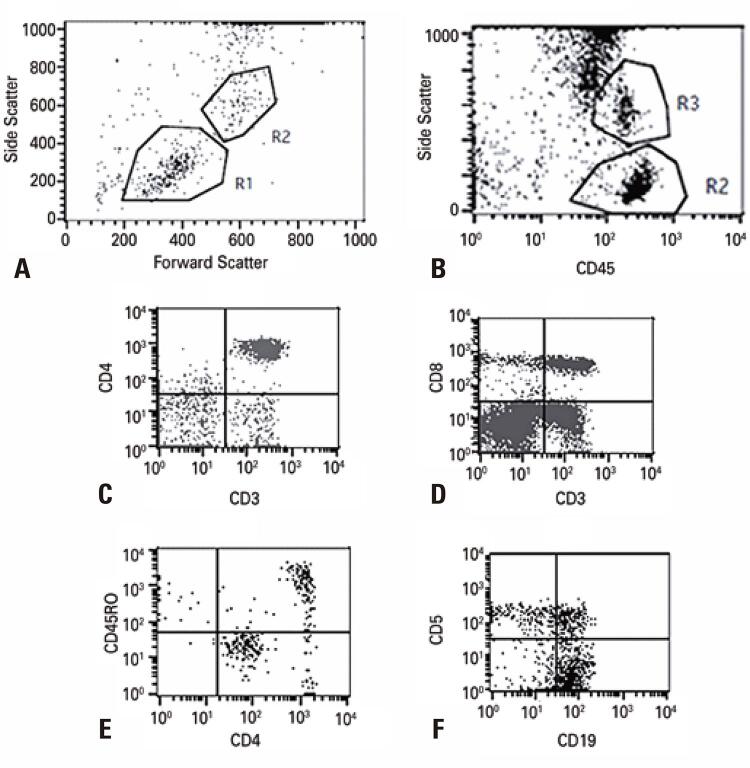
Flow cytometry gating strategy to lymphocyte subsets identification. Lymphocyte (R1) and monocytes (R2) were isolated by forward scatter *versus* side scatter strategy (A) and side scatter *versus* CD45+ (B) CD4+ (C) CD8+ (D) T lymphocytes, memory T CD4+ cells (E) and B lymphocytes (F) were identified by the quadrante that express double positive to the X and Y axis of the dot plots

The gating strategy for B and T cell subsets is shown in figure 1 . First, we gated lymphocytes and monocytes (R1 and R2) identified by a forward scatter *versus* side scatter dot plot and CD45+ *versus* side scatter (R2 and R3) from leukocytes. Subsets of T cells, B cells, and monocytes were evaluated according to the membrane surface markers described in the materials and methods section.

### Lymphocytes

The lymphocyte subsets, median Natural Killer values, and average results for the overall study population are shown in table 1 . The relative values of total T and B lymphocytes are called the overall lymphocyte values.

**Table 1:** Medians and normal ranges for subsets lymphocytes and Natural Killers from an indigenous population in Brazil

Cells	Median (%cells/µL)	Normal range (%cells/µL)
Total T lymphocytes	64.3	(48.7-77.2
CD4+ T lymphocytes	33.4	(18.2-49.8)
CD8+ T lymphocytes	22.6	(12.6-34.3)
CD4+CD8+ (Double positive T cells)	1.4	(0.7-2.6)
Memory CD4+ T lymphocytes	6.2	(3.7-9.2)
B Lymphocytes	10.3	(5.4-17.7)
Regulatory T cells	3.8	(2.5-6.1)
Natural Killers	14.1	(2.4-18.9)

Relative T and B lymphocyte counts were stratified and compared between age groups. We identified two groups (18-35 years old and 36-67 years old) for these comparisons, and the data showed no statistically significant differences.

**Figure 2:**
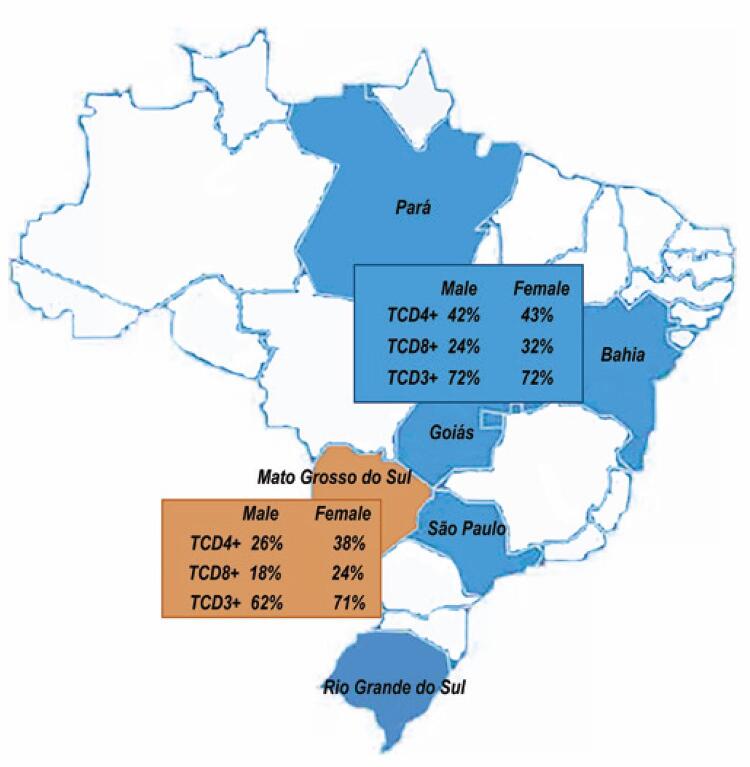
T lymphocytes subsets reference values of indigenous from Mato Grosso do Sul and populations of other states in Brazil

Indigenous females had a higher relative median CD3 T lymphocyte count, CD4 T cell count, and CD8 T cell count than males (p<0.01 for all comparisons). The CD4/CD8 ratio was 1.4 for males and 1.6 for females. The data are shown in figure 2 .

We found no statistically significant difference between the relative median values of lymphocytes and lymphocyte subsets when stratifying individuals according to alcohol intake and smoking. The T- and B-lymphocyte data are shown in table 2 .

**Table 2:** Median for lymphocytes cells and Natural Killers cells based on sex and behavior characteristics of the studied population

	CD3+ T lymphocyte	CD4+ T lymphocytes	CD8+ T lymphocytes	Memory CD4+T cells	B lymphocytes	Regulatory T cells	Natural Killers
Age							
18-35	62	31	22		10.4		
36-67	58	35	27		10.1		
Sex							
Male	62*	26*	18*	5.9	9.8	3.3	15.4
Female	71*	38*	24*	6.3	10.7	3.9	13.9
Smoker							
Yes	64	33	20	5.9	10	3.7	14
No	63	33	23	6.7	10.5	4	14.1
Alcohol use							
Yes	62	30	20	6	9.6	4	13
No	61	32	22	6.4	10.9	4.5	15

* p<0.01.

### Comparison of T lymphocyte subsets among populations from six cities in Brazil

Data on the relative median values for T lymphocyte subpopulations have been published, ^( 20 )^ making it possible to insert them into a new analysis to compare the results obtained. We used the non-parametric Kruskal-Wallis test to evaluate statistical differences among the values presented. We found a statistically significant difference between the values of CD3 T, CD4 T, and CD8 T cells in the cities (p=0.01). Other cells were not evaluated because the study used for comparison with the present study did not measure them. The data it’s shown in table 3 .

**Table 3:** Comparison of T lymphocytes subsets median between populations from cities of Brazil

Cities	TCD4+ median (%cells/µL)	TCD8+ median (%cells/µL)	TCD3+ median (%cells/µL)
Indigenous from Mato Grosso do Sul	33.40 ^†^	22.60 ^†^	64.13 ^†^
Salvador-BA	37.24 ^†^	22.08 ^†^	64.69 ^†^
Belém-PA	36.69 ^†^	19.20 ^†^	60.12 ^†^
Goiânia-GO	38.24 ^†^	24.36 ^†^	65.95 ^†^
Ribeirão Preto-SP	47.14 ^†^	26.20 ^†^	76.16 ^†^
Porto Alegre-RS	43.71 ^†^	24.85 ^†^	71.46 ^†^

Souce: Torres AJ, Angelo AL, Silva MO, Bastos MC, Souza DF, Inocêncio LA, et al. Establishing the reference range for T lymphocytes subpopulations in adults and children from Brazil. Rev Inst Med Trop São Paulo. 2013;55(5):323-8. ^( 19 )^

^†^ Statistical difference (p=0.01) between median values by Kruskal-Wallis non-parametric test.

### Relative class values of monocytes and dendritic cells

We performed immunophenotyping to determine the typical relative median and normal range values for the subsets of monocytes and dendritic cells. Classical, non-classical, and intermediate monocytes are essential markers of inflammatory diseases. The results are summarized in table 4 .

**Table 4:** Median percentage values of monocyte subsets and dendritic cells of the indigenous population from Brazil

Cells	Median (%cells/µL)	Normal range (%cells/µL)
Classical monocytes	83.9	(70.1-90.7)
Non-classical monocytes	7.8	(5.1-8.9)
Intermediary monocytes	7.3	(4.3-7.5)
Dendritic cells	0.18	(0.07-0.31)

## DISCUSSION

Determination of reference ranges for immunological cells provides essential parameters for assessing health conditions within specific populations of individuals. Genetic characteristics such as polymorphisms and environmental and bioavailability factors may vary between individuals and thus demonstrate changes in cell reference values associated with ethnicity. No studies in Brazil have determined reference values for blood cells from indigenous populations.

Indigenous Brazilian residents live in villages with poorer sanitary conditions than in the state capital and other larger cities. Additionally, access to healthy politicians has been limited, and studies evaluating and understanding the physiological parameters of these populations are scarce. ^( 21 )^ The reference range for immunological cells presented in this study is pioneering in Brazil and will contribute to clinical and laboratory evaluations of indigenous populations.

European and Asian studies have indicated an important relationship between immune cell values and ethnicity, ^( 22 , 23 )^ demonstrating variations in median values of B lymphocytes, T lymphocytes, and Natural Killer cells differ in some characteristics. Factors such as stress, nutrition, and infections can alter laboratory test results, ^( 24 )^ increasing the relevance of determining regional reference values for use as medical evaluation parameters.

A study conducted in a healthy Chinese population reported an average of 67.90% for T lymphocytes, 34.1% for CD4+ T cells, and 24.9% for CD8+ T cells. The lymphocyte reference range results of healthy individuals in different countries worldwide present different results associated with ethnic groups, showing the importance of obtaining specific evaluation parameters for different racial patterns. ^( 23 , 25 - 29 )^

The influence of sex on leukocyte subsets was also analyzed in this study. The CD4+/CD8+ ratio was higher in women than in men, which is consistent with results reported in other countries. ^( 29 , 30 - 32 )^ Some studies have suggested that the percentage of T lymphocyte subsets is generally low in men, ^( 30 , 31 )^ while others have found no differences between the sexes. ^( 22 , 32 )^ Additionally, our results showed that the relative number of Natural Killer cells was lower in women than in men, which is consistent with previous reports. ^( 20 , 31 )^ Different levels of sex hormones may underlie the sex differences in lymphocyte subsets, as estrogen levels are higher in women and can block early T cell development in the thymus. ^( 33 )^

An increase in CD4+CD8+ double-positive (DP) T cells in the peripheral blood has been associated with the risk of plasma leakage in some viral diseases ^( 29 )^ and human urogenital cancers, with conflicting data regarding their role. ^( 30 )^ Here, we found a median total DPT of 1.4% of the relative lymphocyte count that requires further investigation because it may represent a value pattern associated with population characteristics or some clinical alteration developed by viral infections.

Smoking affects the Bronchoalveolar Lavage fluid (BAL) cell profiles in different lung diseases, ^( 34 - 38 )^ and reference values for BAL fluid are potential diagnostic markers for interstitial lung diseases. ^( 39 , 40 )^ However, differential peripheral blood cell counts and lymphocyte subpopulations did not differ from the reference intervals between smokers and nonsmokers. ^( 38 )^ In this study, we compared the reference intervals of the relative lymphocyte subset values for indigenous nonsmokers and smokers but did not find a statistical difference between them. However, when we compared the results between individuals who reported alcohol consumption and those who did not, the difference was not statistically significant. Low frequency of alcohol consumption may be the main factor contributing to these results.

Previously, our group published reference range values for T lymphocyte subpopulations in blood donors from five Brazilian states, one from each region of the country, ^( 23 )^ and we found statistically significant differences between the results. When we included the respective results from the present study, a statistical difference was maintained for CD3+ T, CD4+ T, and CD8+ T cells when all states were compared. The immune response is usually modulated by different factors such as nutritional aspects, ethnicity, age, and sex. ^( 28 , 32 )^

Although the differences found in our study were not large enough to preclude the use of the current standard reference, it is important to emphasize regional variations, making it possible for clinicians to define the actual stage of immunodeficiency in our patients more accurately. In figure 2 , we show the T-lymphocyte values of indigenous men and women from this study and five states in Brazil published by our group.

## CONCLUSION

The determination of reference values of immunological cells for Brazilian indigenous populations is a significant advance in the clinical decision-making process owing to the broad ethnic, sex, and behavioral variability characteristics according to the region of the country. The reference range found in our study is important for including indigenous Brazilian individuals with determined immunological parameters to more accurately assess health conditions and define the natural and pathological stages presented by our patients.
